# Genome-wide analysis of *BBX* gene family in Tartary buckwheat (*Fagopyrum tataricum*)

**DOI:** 10.7717/peerj.11939

**Published:** 2021-08-11

**Authors:** Jiali Zhao, Hongyou Li, Juan Huang, Taoxiong Shi, Ziye Meng, Qingfu Chen, Jiao Deng

**Affiliations:** School of Life Sciences, Research Center of Buckwheat Industry Technology, Guizhou Normal University, Guiyang, China

**Keywords:** Regulation, Anthocyanin biosynthesis

## Abstract

BBX (B-box), a zinc finger transcription factor with one or two B-box domains, plays an important role in plant photomorphogenesis, growth, and development as well as response to environmental changes. In this study, 28 Tartary buckwheat *BBX* (*FtBBX*) genes were identified and screened using a comparison program. Their physicochemical properties, gene structures, conserved motifs, distribution in chromosomal, and phylogeny of the coding proteins, as well as their expression patterns, were analyzed. In addition, multiple collinearity analysis in three monocots and three dicot species illustrated that the BBX proteins identified from monocots clustered separately from those of dicots. Moreover, the expression of 11 candidate *BBX* genes with probable involvement in the regulation of anthocyanin biosynthesis was analyzed in the sprouts of Tartary buckwheat during light treatment. The results of gene structure analysis showed that all the 28 *BBX* genes contained B-box domain, three genes lacked introns, and these genes were unevenly distributed on the other seven chromosomes except for chromosome 6. The 28 proteins contained 10 conserved motifs and could be divided into five subfamilies. *BBX* genes of Tartary buckwheat showed varying expression under different conditions demonstrating that FtBBXs might play important roles in Tartary buckwheat growth and development. This study lays a foundation for further understanding of Tartary buckwheat *BBX* genes and their functions in growth and development as well as regulation of pigmentation in Tartary buckwheat.

## Introduction

Zinc finger protein (ZFP) transcription factors are a large class of transcription factors, capable of interacting with DNA, RNA and proteins, and regulate transcription, RNA packaging, apoptosis, protein folding and assembly, among others (Noman et al., 2019). Based on the protein structure, ZFP can be divided into several subfamilies. Among them, BBX (B-box) is a subfamily of zinc finger structural protein family that occurs in all eukaryotes from single-celled to multicellular organisms (Crocco & Botto, 2013; Fang et al., 2019). These proteins contain one or two B-box domains that are involved in protein-protein interaction with some of them having a CCT (CONSTANS, CO-like and TOC1) domain (Wei et al., 2020). The B-box domain contains one or two B-box motifs with about 40 amino acid residues. Based on the consistency and differences in amino acid sequences of the B-box motif, and the specificity of the zinc ion binding site, the B-box domain can be divided into two types: B-box 1 and B-box 2. Both of them are quite conservative. The CCT domain which is involved in transcriptional regulation and nuclear protein transport contains 42∼43 amino acid residues that are also highly conserved (Gangappa & Botto, 2014).

Previous studies have reported that BBX proteins are involved in seedling de-etiolation, by controlling hypocotyl and lateral root growth and cotyledon extension (Crocco & Botto, 2013; Wei et al., 2016). In *Arabidopsis thaliana*, AtBBX4, AtBBX20, AtBBX21, and AtBBX22 promotes light morphogenesis, while AtBBX18, AtBBX19, AtBBX24, AtBBX25, and AtBBX32 inhibits light morphogenesis (Datta et al., 2006); In apple, Bai et al. (2014b) found that one BBX protein MdCOL11 responds to UV-B, and interacts with MdHY5 and thus increases anthocyanin accumulation and coloration in apple pericarp. Therefore, BBX proteins play coordinated and antagonistic roles in the regulation of photomorphogenesis in seedlings. Studies have shown that BBX proteins are closely related to regulatory transcription factors such as HY5 (ELONGATED HYPOCOTYL 5) and COP1 (CONSTITUTIVE PHOTOMORPHOGENIC 1) in the light signaling pathway, and are likely to cross-talk with other pathways (Wang et al., 2011). In addition, as one part of photomorphogenesis, light-induced anthocyanin synthesis is also regulated by BBX proteins. AtBBX21, AtBBX22, and AtBBX23 were reported to be positive regulators of anthocyanin synthesis in Arabidopsis (Xu et al., 2016; Chang, Maloof & Wu, 2011; Zhang et al., 2017b), while AtBBX24, AtBBX25, and AtBBX32 inhibited the biosynthesis and accumulation of anthocyanin (Job et al., 2018; Gangappa et al., 2013; Holtan et al., 2011). AtBBX21 and AtBBX24 can affect the synthesis of anthocyanin by directly interacting with HY5. In tomato, SlBBX20 regulates the synthesis of carotenoids by directly binding to the promoter of the carotenoid biosynthesis key enzyme, PHYTOENESYNTHASE 1 (Xiong et al., 2019). In pear, two B-box proteins, PpBBX18 and PpBBX21, antagonistically regulate anthocyanin biosynthesis *via* competitive association with HY5 in the peel of the fruit (Bai et al., 2019a).

BBX protein has been thoroughly studied in *Arabidopsis thaliana*. The first BBX protein CONSTANS (CO/AtBBX1) found in Arabidopsis interacts with SPA1 (suppressor of phyA-105 1), which contains a coiled helix domain and participates in the flowering control process affected by photoperiod (Laubinger et al., 2006). When R:FR decreases growth density is high and BBX proteins are involved in regulating shade avoidance response. For example, AtBBX19, AtBBX21, and AtBBX22 inhibit the shade avoidance response, while AtBBX18 and AtBBX24 promote the shade avoidance response. The expression of plant hormone related genes involved in shade avoidance response is also regulated by BBX protein (Crocco et al., 2011), BBX protein has been shown to play an important role in hormone signal transduction such as Indole-3-acetic acid (IAA), Gibberellic acid (GA), Abscisic acid (ABA), and Brassinosteroids (BR) (Vaishak et al., 2019). In addition to being involved in plant growth and development, BBX protein can also respond to abiotic stress. For example, AtBBX18 is also involved in heat tolerance response (Wang et al., 2013). AtBBX24 participates in the signal transmission of salt stress and indirectly participates in the molecular pathway related to the improvement of plant salt tolerance (Nagaoka & Takano, 2003). A considerable number of *BBX* genes in apples were up-regulated under osmotic pressure, high salt, low temperature, and ABA (Abscisic acid) treatment (Liu et al., 2018b).

At present, the *BBX* gene has been deeply studied in Arabidopsis, rice, apple among others. Buckwheat as a grain and a medicinal crop, especially Tartary buckwheat has extremely high nutritional and medicinal value. There is no report regarding the *BBX* gene family in buckwheat. The identification and bioinformatics analysis of Tartary buckwheat BBX transcription factors is of great significance to further understand their role in the growth and development, especially in the development of Tartary buckwheat seedlings and anthocyanin synthesis. Combined with public database resources, the authors carried out bioinformatics analysis of the *BBX* gene family in Tartary buckwheat at the genome level, and preliminarily analyzed the chromosome location, classification, phylogeny, and physicochemical properties of the *BBX* gene family, in order to provide a basis for enriching the expression regulation of BBX transcription factors and the function of *BBX* gene family.

## Materials and Methods

### Identification and analysis of *BBX* gene family from Tartary buckwheat

32 BBX protein sequences of Arabidopsis were downloaded from Arabidopsis transcription factor database (https://www.arabidopsis.org/), and Tartary buckwheat genome, predicted coding region sequence (CDS), and protein sequences were downloaded from Tartary buckwheat Genome Project (http://www.mbkbase.org/Pinku1/) (Zhang et al., 2017a). The Arabidopsis BBX proteins were aligned together with the Tartary buckwheat proteins data by Local Blastp (1e−10) of BioEdit software (7.1.9 Version). The preliminary homologous candidate sequences of Tartary buckwheat were obtained. After the repeat sequences were removed from the preliminary candidate sequences, the conserved domains were analyzed by the Conserved Domain Database (CDD) database (https://www.ncbi.nlm.nih.gov/Structure/cdd/cdd.shtml), and the protein sequences without BBX domain were deleted, and the members of Tartary buckwheat BBX transcription factor family were obtained.

The length of coding region sequence, the number of amino acids, the relative molecular weight, isoelectric point, the number of aliphatic amino acid number as well as protein hydrophobicity of Tartary buckwheat BBX family proteins were analyzed by an online Protparam software provided by ExPaSy (https://web.expasy.org/protparam/).

### Synteny analysis and chromosome localization

All the *FtBBX* genes were mapped on 8 chromosomes according to the genome database obtained from Tartary buckwheat Genome Project by TBtools. The syntenic blocks used for constructing a synteny analysis map within the Tartary buckwheat genome and between *Oryza sativa*, *Zea mays, Sorghum bicolor*, *Solanum lycopersicum*, *Fagopyrum tataricum*, and *Arabidopsis thaliana* genomes, were obtained by using BLASTP of TBtools with the reference *E* < 1e−10 and top 10 matches. Syntenic blocks were identified and the collinearity results were visualized using the Multiple Collinear Scan Kit (MCScanX) (Wang et al., 2012) and TBtools software (Chen et al., 2020). The rice genome file downloaded from the MSU Rice Genome Annotation Project Database (http://rice.plantbiology.msu.edu/) (Kawahara et al., 2013), The maize and sorghum genome file downloaded from the plant genome database (http://plantgdb.org). The tomato genome file was downloaded from Sol Genomics Network (https://solgenomics.net/organism/Solanum_lycopersicum/genome). The *Arabidopsis thaliana* genome files are downloaded from Arabidopsis Information Resource (TAIR) database (http://www.arabidopsis.org).

### Gene structure, conserved motif analysis of *FtBBX* genes

The structure of exon and intron of each *FtBBX* gene was analyzed using the TBtools software. Conservative domain analysis was carried out by CDD database. Conserved motifs of each FtBBX protein were identified using the MEME online database (Multiple Em for Motif Elicitation, http://memesuite.org/tools/meme). The maximum motif number was set as 10 and the motif length was set as 6–200 amino acids, and the other parameters were set at default (Bailey & Elkan, 1995).

### Construction of phylogenetic tree

The phylogenetic tree of FtBBXs and AtBBXs proteins were constructed using MEGA7.0 program by Maximum Likelihood (ML) method and bootstrap analysis (1,000 replicates) (Kumar, Stecher & Tamura, 2016), and “JTT+G” was found to be the best ML model using MEGA 7.0 program.

Expression profile analysis of *FtBBX* genes in tissue specificity and response to light and salt stressSince BBX proteins were involved in plant photomorphogenesis and flavonoids biosynthesis (Xu et al., 2018; Gangappa & Botto, 2014; Wang et al., 2011), and BBX protein can also respond to abiotic stress and hormone signals. The expression profiles of *FtBBXs* of Tartary buckwheat seedling in response to different illumination treatment, including dark, far-red light, red light and blue light, were chosen from the RNA-seq data (The sequencing data have been deposited in the National Center for Biotechnology Information (NCBI) database, Accession number: SRP157461) by Zhang et al. (2019) (in whose paper, data of Supplemental Data, Table S2 was used, https://onlinelibrary.wiley.com/doi/abs/10.1111/pce.13470). The expression profiles of the *FtBBX* genes in each sample were collected at 0 and 24 h after salinity treatment using RNA-seq data (The sequencing data have been deposited in the National Center for Biotechnology Information (NCBI) database, accession number: SRR6068977, SRR6068978) recently published by Wu et al. (2017) from Tatary buckwheat plants. In addition, we analyzed the expression profiles of the 28 *FtBBX* genes in different tissues using RNA-seq data (The sequencing data have been deposited in the National Center for Biotechnology Information (NCBI)database, Accession number: Root SRR5433734, Stem SRR5433731, Leaf SRR5433730, Flower SRR5433732) recently published by Zhang et al. (2017a) and Li et al. (2019). The RNA-seq data processing procedure refer to the transcriptome data processing method in Liu et al. (2018a) And the FPKM (Fragments Per Kilobase of transcript sequence per Millions base pairs sequenced, at least one sample must have had fragments per kilobase of transcript per million mapped reads (FPKM) ≥ 0.5) values of FtBBXs were used to construct heatmap using the TBtools software.

### FtBBX genes that regulates anthocyanin biosynthesis in Tartary buckwheat

Six proteins (AtBBX21, AtBBX22, AtBBX23, AtBBX24, AtBBX25, and AtBBX32) which have regulatory effect on anthocyanin synthesis in Arabidopsis ((Bai et al. 2019b) were selected as the search sequences, and the FtBBX which may regulate anthocyanin synthesis were screened out from 28 FtBBX sequences. Then ClustalW program was used for multiple sequence alignment of these related BBX proteins. A phylogenetic tree based on BBX proteins sequence was constructed by using MEGA7.0 program neighbor-joining method and bootstrap analysis (1,000 replicates) (Kumar, Stecher & Tamura, 2016).

In order to further identify the *FtBBX* gene that might be involved in anthocyanin synthesis, we analyzed the expression correlation between *FtBBX* gene and some genes involved in the synthesis of flavonoids based on RNA-seq expression data by Zhang et al. (2019). The correlation was obtained in Excel and the TBtools was used to draw the heat-map of correlation coefficients.

### Plant materials and anthocyanins measurement

The Tartary buckwheat cultivar ‘Jinqiao 2’ (‘JQ 2’) was used in this study and germination was performed based on the paper bed germination method. They were then placed in an artificial climate box with the temperature maintained at 26 °C and humidity 70%. First, they were incubated in the dark for 2 days and then followed by light treatment for 0, 6, 12 and 24 h. The stems of the sprouts at these five periods of light treatment were collected and snap-frozen in liquid nitrogen and immediately stored at −80 °C for further use.

The anthocyanin was extracted from the samples mentioned above according to the previous report (Rahim, Busatto & Trainotti, 2014) with slight modification. Briefly, 1 g sample was weighed and powered using liquid nitrogen. The extraction was achieved by adding four mL methanol containing 1% (v/v) HCl and incubated for 24 h at 4 °C. Afterwards, the mixture was centrifuged at 12,000 rpm for 10 min. The supernatant was then collected and the absorbance value was measured at 530 nm and 657 nm, respectively. The anthocyanin content was obtained by the formula Q_anthocanin_ =(A_530_ − 0.25 × A_657_) × M^−1^ (M represented the fresh weight of the sample). Three biological repeats for each sample were analyzed.

### RNA extraction and qRT-PCR analysis

Total RNAs from seedling tissues mentioned above were extracted by GREENspin Plus Plant RNA kit (ZoManBio, Beijing, China) according to the manufacturer’s protocol. And cDNA was synthesized using a First Strand cDNA Synthesis Kit (TOYOBO, Japan). The expression level of 11 *BBX* genes, which were speculated to be involved in regulation of anthocyanin biosynthesis, in the sprouts after irradiation for 0, 6, 12 and 24 h were analyzed by qRT-PCR. A 20 μL reaction system was used which contained 10 μL 2 × iQ^™^ SYBR Green Super mix (Bio-Rad, Irvine, CA, USA), 0.5 μL 10 μM of specific forward and reverse primer, respectively, 1 μL containing about 100 ng cDNA. qRT-PCR was carried out on the C1000^™^ thermal cycler coupled with a CFX96^™^ detection module (Bio-Rad) with the following program: 95 °C, 3 min; 40 cycles of 95 °C for 10 s, 55 °C for 30 s and 72 °C for 10 s. The primers sequences are listed in Supplemental File 1.

The relative expression of these genes was calculated using 2^−∆∆Ct^ method, and performed with triplicate biological repeats. To analyze significant differences, one-way ANOVA was conducted using IBM SPSS Statistics 22.0.

## Results

### Identification and distribution analysis of the *BBX* genes in Tartary buckwheat genome

A total of 28 putative *BBX* genes were identified in Tartary buckwheat which were named as *FtBBX1* to *FtBBX28*. Detailed information including gene name, gene ID, intron number, protein length, MW (molecular weight), theoretical pI (isoelectric point), aliphatic index, and GRAVY (grand average of hydropathicity) of FtBBXs is listed in Table 1. *FtBBX1*, *FtBBX8*, and *FtBBX21* genes contained no intron, and the number of introns in other genes ranges from one to six. Among them, nine members contained only one intron and eight genes contained two introns. *FtBBX11* and *FtBBX12* comprised the largest number of introns, six and five, respectively. The 28 FtBBX proteins had diverse amino acids (aa) and molecular weight (MW) with the number of aa ranging from 114 (FtBBX1) to 419 (FtBBX7) and MW ranged from 12.78 kDa to 46.51 kDa. Theoretical isoelectric points (pI) of these FtBBX proteins varied from 4.20 (FtBBX21) to 8.22 (FtBBX10) and the value of the aliphatic index ranged from 52.26 (FtBBX9) to 95.88 (FtBBX1), indicating a varying thermostability of this family of proteins. The GRAVY of FtBBX protein was less than zero except that of FtBBX1 (0.118), implying that the majority of FtBBX proteins were hydrophilic proteins.

**Table 1:** Basic information of *BBX* family genes in Tartary buckwheat.

Gene name	Gene ID	Intron number	Protein/aa	MW (Da)	pI	Aliphatic index	GRAVY
*FtBBX1*	FtPinG0008534900.01.T01	0	114	12781.83	6.09	95.88	0.118
*FtBBX2*	FtPinG0006549100.01.T01	2	253	28192.96	5.48	84.39	−0.349
*FtBBX3*	FtPinG0008621100.01.T01	3	327	35128.98	6.06	60.03	−0.499
*FtBBX4*	FtPinG0006138300.01.T01	1	254	27914.32	5.51	71.38	−0.345
*FtBBX5*	FtPinG0008738000.01.T01	1	356	40364.42	7.17	66.29	−0.79
*FtBBX6*	FtPinG0001521000.01.T01	2	264	29661.51	5.1	79.77	−0.403
*FtBBX7*	FtPinG0003751600.01.T01	3	419	45833.02	4.96	67.26	−0.455
*FtBBX8*	FtPinG0000904300.01.T01	0	258	28354.12	6.92	75.93	−0.331
*FtBBX9*	FtPinG0007958700.01.T01	2	243	26651.43	6.11	52.26	−0.563
*FtBBX10*	FtPinG0007693900.01.T01	1	124	13951.98	8.22	84.19	−0.328
*FtBBX11*	FtPinG0008082900.01.T01	6	414	46512.47	6.13	58.7	−0.713
*FtBBX12*	FtPinG0000614000.01.T01	5	397	43516.39	5.42	57.81	−0.627
*FtBBX13*	FtPinG0005709400.01.T01	1	192	20487.53	4.52	64.06	−0.447
*FtBBX14*	FtPinG0003122700.01.T01	1	376	42553.85	6.05	68.51	−0.668
*FtBBX15*	FtPinG0007743000.01.T01	4	205	22657.56	7.57	65.27	−0.619
*FtBBX16*	FtPinG0009564600.01.T01	1	378	41234.24	5.66	72.04	−0.334
*FtBBX17*	FtPinG0002738900.01.T01	2	226	25300.69	5.96	70.4	−0.477
*FtBBX18*	FtPinG0008999600.01.T01	2	371	41072.56	5.91	61.29	−0.631
*FtBBX19*	FtPinG0006024400.01.T01	4	414	45005.81	4.98	61.55	−0.519
*FtBBX20*	FtPinG0003531700.01.T01	1	349	38323.08	6.01	75.73	−0.255
*FtBBX21*	FtPinG0002269700.01.T01	0	135	14728.83	4.2	57.85	−0.796
*FtBBX22*	FtPinG0003402900.01.T01	1	376	40829.45	5.52	66.3	−0.432
*FtBBX23*	FtPinG0009261700.01.T01	2	283	30503.38	5.38	75.83	−0.225
*FtBBX24*	FtPinG0005206600.01.T01	2	239	26513.04	5.03	80.5	−0.338
*FtBBX25*	FtPinG0009408600.01.T01	2	327	36302.66	6.13	65.93	−0.522
*FtBBX26*	FtPinG0005054800.01.T01	4	168	18795.14	6.18	61.61	−0.637
*FtBBX27*	FtPinG0006374000.01.T01	3	391	43083.76	4.94	70.84	−0.556
*FtBBX28*	FtPinG0004072200.01.T01	1	281	31151.97	7.93	69.79	−0.371

**Note:**

aa, amino acid residues; MW, molecular weight; pI, theoretical isoelectric point; GRAVY, grand average of hydropathicity.

**Figure 1:**
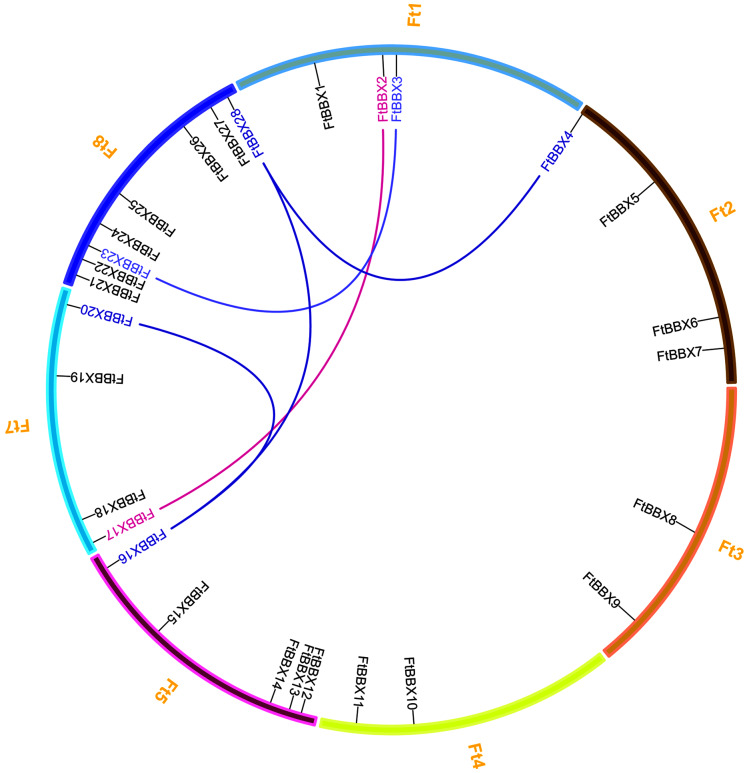
Chromosome distribution and segmental duplication of Tartary buckwheat *BBX* genes. Five pairs of the segmental duplicated genes are indicated in a different color and are connected by lines.

There are eight chromosomes in the Tartary buckwheat genome (Zhang et al., 2017a). The 28 *FtBBX* genes were distributed unevenly throughout the seven chromosomes except for chromosome 6 (Fig. 1). Among them, chromosome 8 had the highest number of *FtBBX* genes (8), while five *FtBBX* genes were localized on chromosome 5. Chromosomes 2 and 7 had four *FtBBX* genes each while chromosomes 3 and 4 had two *FtBBX* genes each (Fig. 1). Tandem duplication and segmental duplications occur frequently in gene families’ evolution and expansion. Five pairs of duplicated segments (*FtBBX2*/*FtBBX17*, *FtBBX3*/*FtBBX23*, *FtBBX4*/*FtBBX28*, *FtBBX16*/*FtBBX20*, and *FtBBX16*/*FtBBX28*) in *FtBBX* gene family were identified within the Tartary buckwheat genome. The result suggested that segmental duplication played an important role in the amplification of *BBX* gene family members in the Tartary buckwheat genome.

### Phylogeny, multiple collinearity relationship, conserved motifs and gene structure analysis of *FtBBXs*

**Figure 2:**
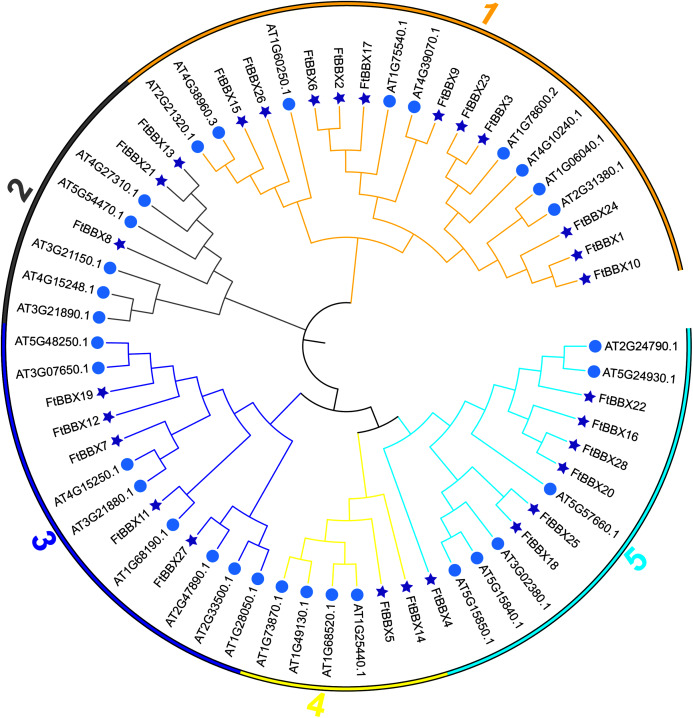
Phylogenetic tree of *BBX* genes from Tartary buckwheat and Arabidopsis.

**Figure 3:**
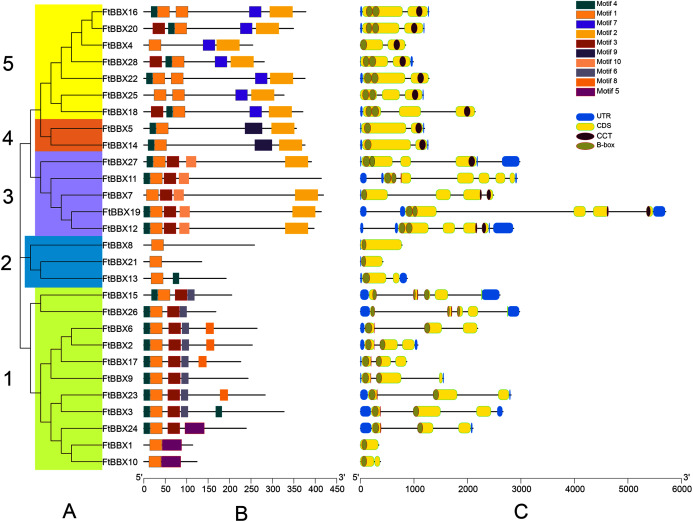
Phylogeny, conserved motifs and gene structure of *FtBBXs* gene family. (A) Phylogenetic relationship of the FtBBX proteins. The five subfamilies (1–5) are denoted by different colors; (B) conserved motifs of FtBBX proteins. Each motif is marked with a different color, and the black line represents the non-conservative sequence. The size of each motif is proportional to the scale; (C) structure of *FtBBX* genes. Exons and domains are indicated by color frames, introns are represented by black lines.

All FtBBX proteins could be classified into five subgroups based on the phylogenetic analysis of FtBBXs and AtBBXs (Fig. 2), as well as the cluster analysis of FtBBXs protein sequences (Fig. 3A), which was consistent with the previous study in grapevine and Arabidopsis (Wei et al., 2020; Gangappa & Botto, 2014; Khanna et al., 2009).

FtBBXs clustering in the same group possessed similar conserved motifs and their genes had similar structure (Fig. 3). Subfamily 1 contained the largest number of FtBBX proteins with 11 members, most of the proteins contained motifs 1, 3, 5 and 7, with several of them containing motif 8 or 5 (Figs. 3A and 3B). Most of the subfamily1 genes contained two B-box domains (Fig. 3C). Subfamily 5 contained the second most number of members (7), with all of them having motifs 1, 2, and 7, while three of them (FtBBX16, FtBBX22, and FtBBX25) contained two motif 1, and several members contained motif 3 and motif 4. Except for *FtBBX4*, which only had one B-box and one CCT conserved domains, the other six *FtBBX* genes had two B-box and one CCT conserved domains (Fig. 3). Subfamily 3 enriched five members. It is only *FtBBX11* that lacked motif 2, and FtBBX 7 which was lacking motif 4, the other three FtBBX proteins contained five types of motif, including motif 1, 3, 4, 5, and 7. Except for *FtBBX7*, the other four genes had two B-box domains. Additionally, most of them also contained the CCT domain (Fig. 3). Three members (FtBBX8, FtBBX21, and FtBBX8) composed subfamily 2, which contained less domain but had a specific motif (motif 7) (Fig. 3). By contrast, subfamily 4 was the smallest one which was composed of only two members. Subgroups 3, 4, and 5 had a close genetic relationship and shared a common parent according to the phylogenetic analysis, predicting that these proteins may have similar functions (Fig. 3A).

**Figure 4:**
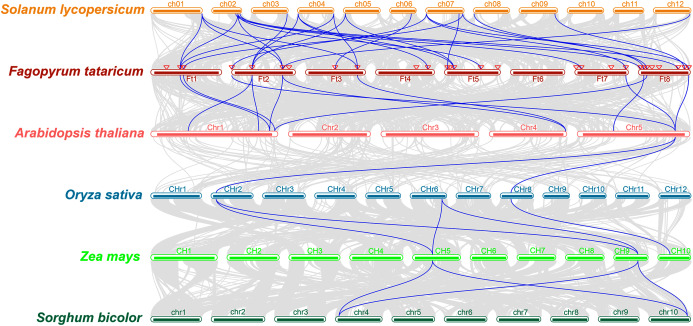
Collinearity relationship of six species genes.

The study of collinearity relationship can provide information for homologous pairs between species. Between rice and pineapple *CPK* genes, four pairs of syntenic orthologous genes were identified by collinearity analysis, indicating that these genes might be derived from the same ancestor of rice and pineapple (Zhang et al., 2020). A multiple collinearity analysis of six species which included monocots (*Oryza sativa*, *Zea mays*, and *Sorghum bicolor*) and dicots (*Solanum lycopersicum*, *Fagopyrum tataricum*, and *Arabidopsis thaliana*) was created using TBtools software. The results (Fig. 4) showed that there were more homologous pairs between dicotyledonous species, and there were fewer homologous pairs between monocotyledons and dicotyledons. In dicots, 15 genes in *Solanum lycopersicum* were linked to 19 genes in *Fagopyrum tataricum*. 10 *BBX* genes in *Fagopyrum tataricum* corresponded to seven genes in *Arabidopsis thaliana*. In monocots, three genes in *Oryza sativa* corresponded to three genes in *Zea mays*, 2 genes in *Zea mays* corresponded to two genes in *Sorghum bicolor*. However, only one gene in *Arabidopsis thaliana* linked to two genes in *Oryza sativa*, indicating that *BBX* genes were conserved in dicots, but there was no obvious relationship between monocotyledons and between monocots and dicot species.

In addition, many *FtBBX* genes had their one-to-one corresponding orthologs between species. However, some *FtBBX* genes had two or more orthologs. For example, *FtBBX2* (on chromosome 1) corresponded with *Solyc01g110370.4.1* (on chromosome 1), *Solyc02g084420.3.1* (on chromosome 2), *Solyc04g081020.3.1* (on chromosome 4) and *AT1G75540.1* (on chromosome 1); *FtBBX5* (on chromosome 2) corresponded with *Solyc03g119540.3.1*, *Solyc04g007210.3.1* (on chromosome 4), *Solyc05g009310.3.1* (on chromosome 5), *AT1G25440.1* and *AT1G68520.1* (on chromosome 1); *FtBBX6* (on chromosome 2) corresponded with *Solyc01g110180.4.1* (on chromosome 1), *Solyc02g084420.3.1* (on chromosome 2), *Solyc12g089240.2.1* (on chromosome 12), *AT1G75540.1* (on chromosome 1) and *AT4G39070.1* (on chromosome 4). Moreover, there were several *BBX* genes in *Arabidopsis thaliana* that had 2 or more duplicated orthologs in *Fagopyrum tataricum*. *AT1G75540.1* (on chromosome 1) corresponded with *FtBBX2* (on chromosome 1) and *FtBBX6* (on chromosome 2); *AT1G78600.1* (on chromosome 1) corresponded with *FtBBX3* (on chromosome 1) and *FtBBX23* (on chromosome 8); *AT5G57660.1* (on chromosome 5) corresponded with *FtBBX4* (on chromosome 2), *FtBBX20* (on chromosome 7) and *FtBBX28* (on chromosome 8). This result offers information about BBX genes’ duplications among species.

### The expression profiling of *FtBBX* genes in tissues-specific and response to light and salt stress

It’s reported that BBX proteins play a vital role in plant growth and developmental processes, especially in seeding photomorphogenesis (Gangappa & Botto, 2014). We investigated the transcript expression patterns at the different developmental tissues and under different treatments to study the biological roles of *FtBBX* genes in the Tartary buckwheat growth and development, based on the published Supplemental Data sets (Zhang et al., 2019; Wu et al., 2017; Zhang et al., 2017a; Li et al., 2019). The expression data of these *FtBBX* genes are presented in the form of a heat map that was constructed using HeatMap IIustructor program of TBtools software based on the Log2(FPKM) value of genes.

**Figure 5:**
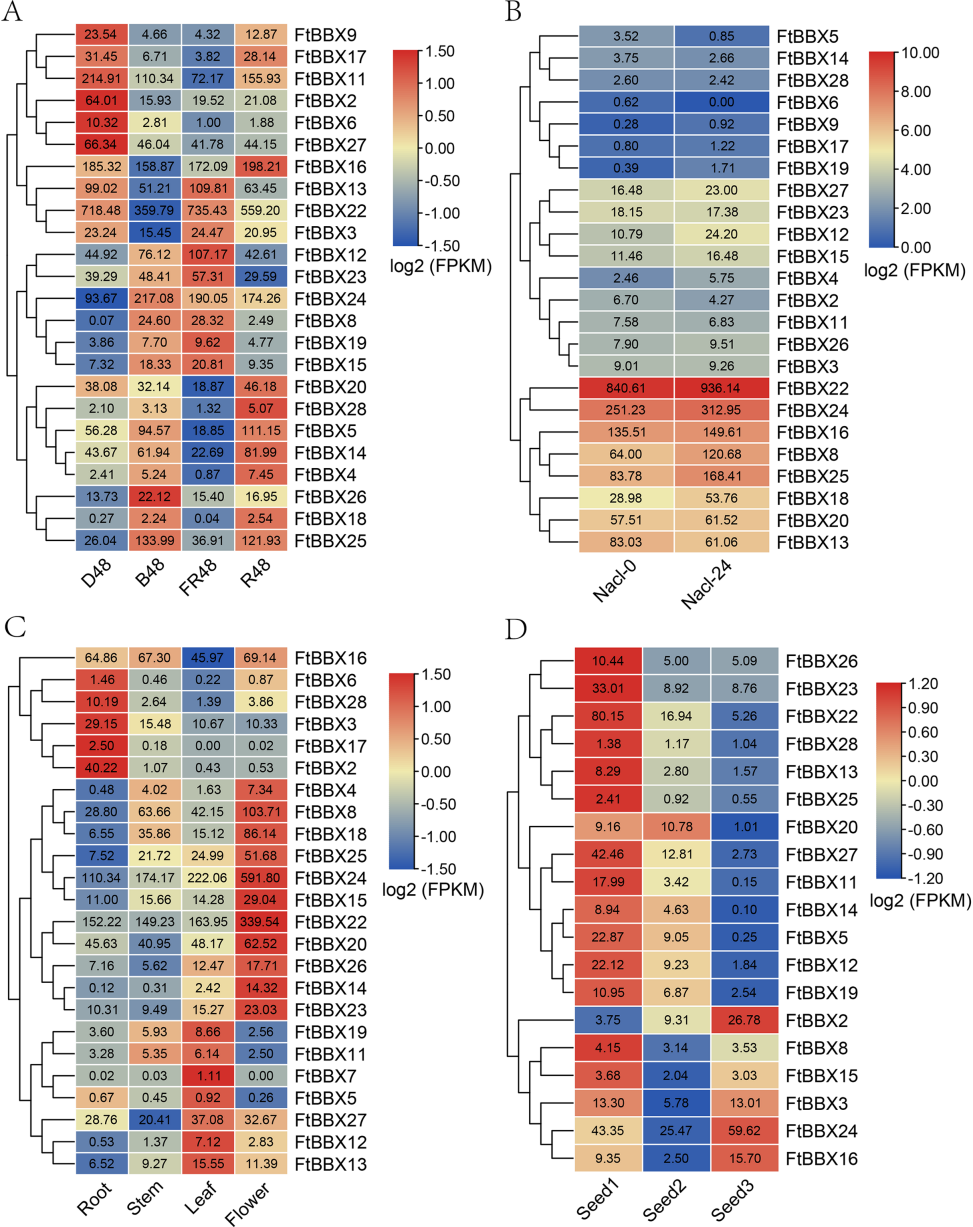
The expression profile of *FtBBX* genes in Tartary buckwheat. (A) The expression patterns of ****Tartary buckwheat seedlings under different light treatment for 48 h including dark (D48), far-red light (FR48), blue light (B48), and red light (R48); (B) the expression patterns of Tartary buckwheat seedlings at 0 and 24 h after salinity treatment; (C) the expression patterns of Tartary buckwheat flowers, roots, leaves, and young stems at 10-week-old under natural light; (D) the expression patterns of Tartary buckwheat seeds of Tartary buckwheat at 12 day, 15 day, and 21 day after full bloom. Numbers in cells are the expression values (FPKM) of genes.

The transcript expression patterns (Fig. 5A) of 28 *FtBBX* genes were investigated under different light treatment for 48h including dark (D48), far-red light (FR48), blue light (B48), and red light (R48) using the published Supplemental Data sets (Zhang et al., 2019). Except for *FtBBX1*, *FtBBX7*, *FtBBX10*, and *FtBBX21*, other genes were expressed in all samples, but the expression patterns of each gene were different. *FtBBX22* had the highest expression level in all 4 samples with its FPKM value ranging from 359.79 to 735.43, which indicated that this gene may be unrelated to the light response signals, but may play an important role during Tartary buckwheat seedlings process. *FtBBX9*, *FtBBX17*, *FtBBX11*, *FtBBX2*, *FtBBX6*, and *FtBBX27* expressed higher in D48 seedlings than those in other illumination conditions. *FtBBX6* expressed higher in D48 seedlings than those in other illumination conditions. *FtBBX4* had a slightly higher expression level in R48 seedlings and *FtBBX19* expressed higher in FR48 and B48 seedlings. *FtBBX18* and *FtBBX28* showed no significant differential expression among the four samples. To explore the mechanisms of *FtBBX* genes response to the salt stresses, we analyzed the expression profiles of the 28 *FtBBX* genes in each sample collected at 0 and 24 h after salinity treatment using RNA-seq expression data recently published by Wu et al. (2017) from Tartary buckwheat plants. Similarly, other genes were expressed except *FtBBX1*, *FtBBX7*, *FtBBX10*, and *FtBBX21*. The results (Fig. 5B) showed that most genes having a low expression level, indicating that most *BBX* genes are not salt responsive. It is noteworthy that the expression values of *FtBBX12*, *FtBBX4*, *FtBBX8*, and *FtBBX25* were significantly changed after salt stress treatment. In particular, the expression levels of *FtBBX8* and *FtBBX25* were 2 times higher than those of the control group after salt stress treatment. It was speculated that *FtBBX8* and *FtBBX25* might play an important role in the response of Tartary buckwheat to salt stress.

To investigate the functional roles of *FtBBX* genes in the Tartary buckwheat genome, we analyzed the expression profiles of the 28 *FtBBX* genes in different tissues using RNA-seq expression data recently published by Zhang et al. (2017a). Similar to the results above, *FtBBX1*, *FtBBX9*, *FtBBX10*, and *FtBBX21* showed no expression, and *FtBBX7* only had a slight expression value in leaves. The results (Fig. 5C) showed that the *BBX* genes were expressed in different tissues in Tartary buckwheat. Some genes showed preferential expression across the detected tissues. Remarkably, *FtBBX24* showed a high expression level in flowers while a low expression level in root, stem, and leaf. *FtBBX8*, *FtBBX18*, *FtBBX25*, *FtBBX15*, *FtBBX22*, *FtBBX20*, and *FtBBX23* also had high expression level in flower and showed similar expression pattern. On the contrary, *FtBBX2* displayed a high expression level in root while barely any expression in stem, leaf, and flower. *FtBBX6*, *FtBBX28*, *FtBBX3*, and *FtBBX17* also had high expression levels in root and showed similar expression patterns, indicating they might participate in the regulation of flower color formation in the Tartary buckwheat.

In addition, we analyzed the expression profiles of the 28 *FtBBX* genes in seeds harvested at 12, 15, and 21 days after full bloom, which corresponding to the initial filling stage (Seed1), peak filing stage (Seed2), and initial maturity stage (Seed3) (Li et al., 2019). According to the result (Fig. 5D), nine *BBX* genes including *FtBBX1*, *FtBBX4*, *FtBBX6*, *FtBBX7*, *FtBBX9*, *FtBBX10*, *FtBBX17*, *FtBBX18*, and *FtBBX21* were also not expressed during seed development. Most genes such as *FtBBX23*, *FtBBX22*, *FtBBX27*, and *FtBBX11* were expressed at a relatively high level at the initial filling stage and a relatively low level at the peak filing stage and the initial maturity stage. It was speculated that these genes played important regulatory roles at the early stage of seed development. Interestingly, the high expression levels of *FtBBX22* in different developmental tissues and under different treatment conditions indicated that the gene might play an important role in the development of Tartary buckwheat, especially in response to hormones.

### Potential BBX proteins involved in anthocyanin biosynthesis in Tartary buckwheat and their expression profile under light treatment

**Figure 6:**
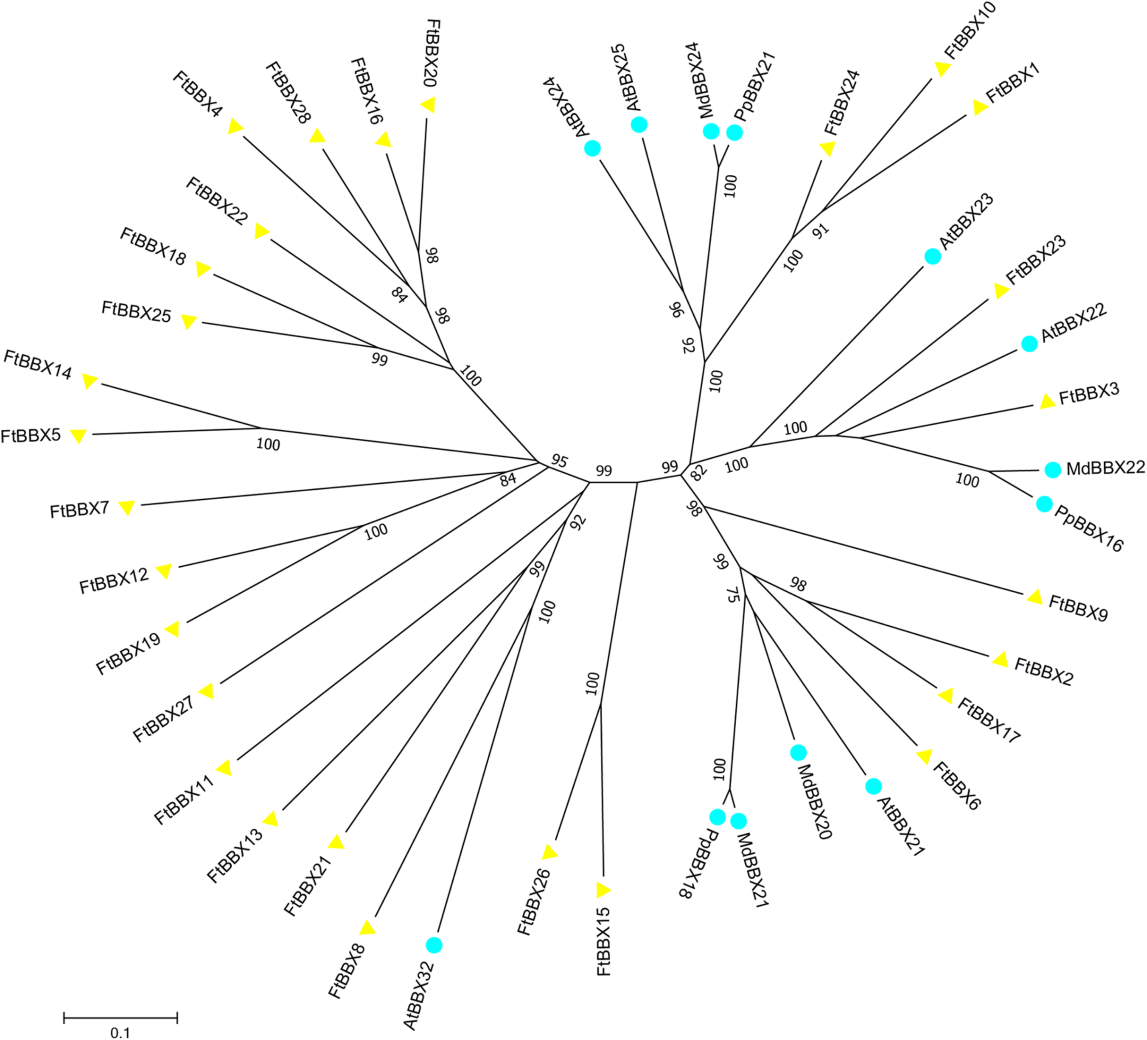
Phylogenetic tree of BBX homologues that regulate anthocyanin biosynthesis. AtBBX21 (*Arabidopsis thaliana*, NP_177686.1); AtBBX22 (*Arabidopsis thaliana*, NP_001185428.1); AtBBX23（*Arabidopsis thaliana*, NP_192762.1); AtBBX24 (*Arabidopsis thaliana*, NP_172094.1); AtBBX25 (*Arabidopsis thaliana*, NP_565722.1); AtBBX32 (*Arabidopsis thaliana*, NP_188752.1); MdBBX22 (*Malus domestica*, XP_008348545.2); MdBBX21 (*Malus domestica*, XP_008387988.1); MdBBX24 (*Malus domestica*,NP_001315848.1); MdBBX20 (*Malus domestica*,XP_008382325.2); PpBBX18 (*Pyrus pyrifolia*, XP_009335579.1); PpBBX21 (*Pyrus pyrifolia*, XP_009342646.1); PpBBX16 (*Pyrus pyrifolia*, XP_009376736.1).

Previous studies suggested that some BBX proteins were involved in anthocyanin production, including the AtBBX21, AtBBX22, and AtBBX23 from Arabidopsis (Xu et al., 2016; Chang, Maloof & Wu, 2011; Zhang et al., 2017b), MdBBX20, MdBBX21, and MdBBX22 from apple (Fang et al., 2019; An et al., 2019), PpBBX16, PpBBX18 from red pear (Bai et al., 2019a, 2019b) were reported to positively regulate anthocyanin biosynthesis. However, AtBBX24, AtBBX25, AtBBX32 (Job et al., 2018; Gangappa et al., 2013; Holtan et al., 2011), MdBBX24 (An et al., 2019) as well as PpBBX21 (Bai et al., 2019b) were identified as negative regulators in anthocyanin accumulation. In the phylogenetic tree, both the positive and negative regulators were divided into two branches, respectively (Fig. 6).

Fifteen FtBBX proteins were obtained after the construction of the phylogenetic tree with those BBX proteins mentioned above and all FtBBX proteins (Fig. 6), which indicated that these 15 FtBBX proteins may be also involved in the regulation of anthocyanin biosynthesis in Tartary buckwheat. Among them, six FtBBX proteins including FtBBX2, FtBBX3, FtBBX6, FtBBX7, FtBBX9, and FtBBX23 were clustered together with those proteins that promote anthocyanin synthesis, therefore, they were speculated to possess similar function. Meanwhile, other five FtBBX members (FtBBX8, FtBBX10, FtBBX15, FtBBX24, and FtBBX26) were presumed to negatively regulate anthocyanin biosynthesis due to their phylogenetic proximity to AtBBX24, AtBBX25, MdBBX24, and AtBBX32 (Fig. 6).

**Figure 7:**
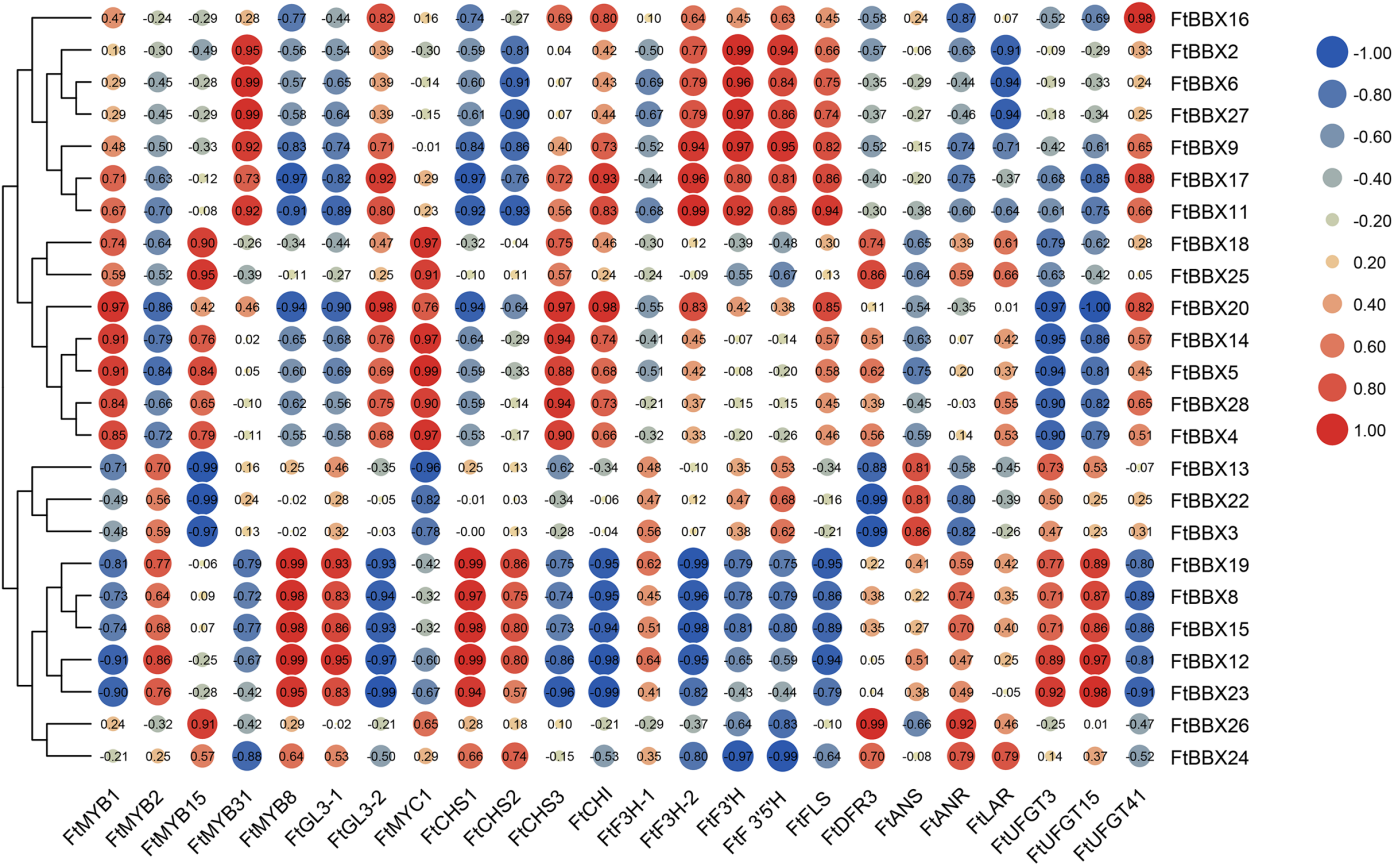
Heat-map of correlation coefficients between *FtBBX* and flavonoids synthesis genes in Tartary buckwheat seedlings under different light conditions. The figures are correlation coefficients and the size of the circle indicates the strength of the correlation.

In order to further identify the *FtBBX* gene which may be involved in anthocyanin synthesis, we analyzed Gene Coexpreesion between *FtBBX* gene and flavonoids-related genes (Bai et al., 2014a; Luo et al., 2018; Sun et al., 2020; Huang et al., 2019; Li et al., 2019; Zhou et al., 2016; Yao et al., 2019) based on the transcriptome data of buckwheat seedlings under different light conditions (Fig. 7). In addition to the significant positive correlation between the *FtBBX3* and the *FtANS* only, four genes, *FtBBX2*, *FtBBX6*, *FtBBX9*, and *FtBBX17*, all showed a positive correlation with *FtMYB31*, *FtF3H-2*, *FtF3′H*, and *FtF3′5′H*. In addition, *FtBBX9* and *FtBBX17* also showed a positive correlation with *FtCHI*, *FtFLS*, and *FtUFGT41*. Moreover, *FtBBX23* was positively correlated with *FtMYB2*, *FtMYB8*, and *FtGL3-1*, also positively correlated with anthocyanin synthesis structural genes *FtCHS*, *FtUFGT3*, and *FtUFGT15*. *FtBBX24* exhibited a negative correlation with *FtMYB31*, *FtF3H-2*, *FtF3′H*, and *FtF3′5′H*.

**Figure 8:**
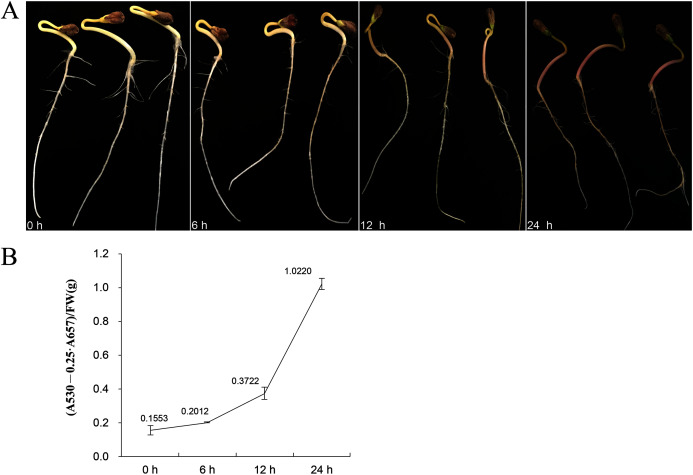
(A) Seedlings of ‘Jinqiao 2’ under light treatment for 0, 6, 12, and 24 h and (B) the total anthocyanin content of these four samples.

Eleven genes were selected for qRT-PCR expression analysis based on the above results. The expression level of these 11 *FtBBX* genes in seedlings of the cultivar ‘Jinqiao 2’ under light treatment for 0, 6, 12 and 24 h were analyzed by qRT-PCR. The total anthocyanins contents of these samples were also measured. The results showed that there was no pigment accumulation in the seedlings grown under dark conditions, but the anthocyanin accumulated when they were grown under light with the main accumulation occurring in the stem tissues (Fig. 8A). The anthocyanin content increased with the duration of light exposure, reaching the maximum at 24 h time point (Fig. 8B, Supplemental File S2).

**Figure 9:**
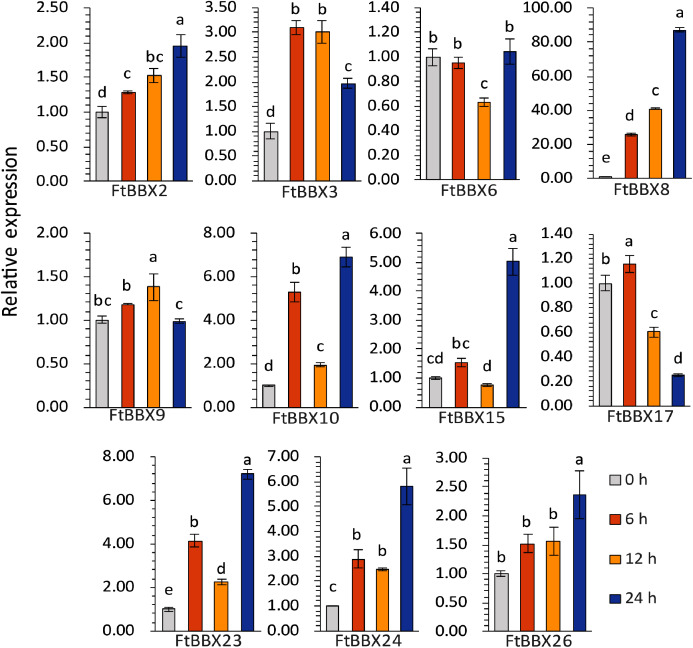
The expression profiles of 11 *FtBBX* genes in Tartary buckwheat seedlings under different illumination treatment time analyzed by qRT-PCR. The lowercase letters above the bar chart indicated significant differences determined by one-way ANOVA, and different letters represented significant differences (*p* < 0.05); Data are means ± SD (*n* = 3).

The expression levels of most genes in Tartary buckwheat seedlings treated with different light times showed significant difference (Fig. 9, Supplemental File 3). Among them, *FtBBX2, FtBBX8*, and *FtBBX26* gradually increased in expression level under light treatment (Fig. 9), which was consistent with the accumulation of anthocyanins. Hence, these three *FtBBX* genes were perceived to be probable positive regulators of anthocyanin biosynthesis in Tartary buckwheat. Although the expression level of *FtBBX3*, *FtBBX10*, *FtBBX15*, *FtBBX23*, and *FtBBX24* didn’t continue to increase in seedlings under light treatment, their expression levels were up-regulated compared with those in the darkness. Therefore, they were also thought to promote anthocyanin accumulation in seedlings when exposed to light. While *FtBBX17* expressed with a slight increase in the seedlings under light treatment for 6 h, then began to decline, and the final expression level was only 1/10 of that at 0 h, which seemed nearly negatively correlating with anthocyanin accumulation. Thus, it’s speculated that *FtBBX17* may inhibit anthocyanin biosynthesis (Fig. 9). In addition, the expressions of *FtBBX6* and *FtBBX9* showed no significant differences among these four samples, which suggested that they may not be responsive to light.

## Discussion

Twenty-eight *BBX* family sequences were identified in Tartary buckwheat, which is similar with the number of *BBX* genes in Arabidopsis (32) and rice (30). Meanwhile, the classification of Tartary buckwheat *BBX* family also has a high similarity with that in Arabidopsis by analysis of phylogenetic tree, gene structure, conserved motifs, indicating that the *BBX* genes remained relatively intact during the plant evolution process. Besides, the results of collinearity analysis showed that the *BBX* gene between the dicots had better homology, and there was a significant difference in the genetic relationship between the dicots and the monocots.

There are some specific transcription factors in *BBX* family. These transcription factors combine with other protein to form a complex, BBX21, BBX22, BBX24 and BBX25 have been reported to genetically interact with HY5 in Arabidopsis (Datta et al., 2007, 2008; Chang et al., 2008; Jiang et al., 2012; Gangappa et al., 2013*)*, and where the B-box domain can form a heterodimer in the BBX protein family or with other proteins (Gangappa et al., 2013). For example, in pear, PpBBX18 physically interacted with transcription factor ELONGATED HYPOCOTYL 5 (HY5) to form the PpHY5–PpBBX18 active transcription activator complex, and subsequently induce *PpMYB10* gene expression and finally activate anthocyanin accumulation in the pee of pear fruit (Bai et al., 2019a), while another BBX protein, PpBBX21, can interact with PpHY5 and PpBBX18 to hinder the formation of the activator complex, and thus inhibiting anthocyanin biosynthesis (Bai et al., 2019a).

In plants, flavonoids are synthesized by phenylpropane, which can be divided into different kinds of derivatives, such as anthocyanins and procyanidins (Lepiniec et al., 2006). The biosynthesis of anthocyanins includes a series of enzymes encoded by structural genes and is relatively conserved among plant species (Qian et al., 2014). Transcription of the genes encoding these enzymes is regulated by the MYB-bHLH-WD40 (MBW) protein complex, HY5, and BBX (Ramsay & Glover, 2005; Bai et al., 2019a). As an important member of zinc finger structural transcription factors, the *BBX* gene family is widely involved in plant growth, development, and environmental response (Gangappa & Botto, 2014). Importantly, the R2R3-MYBs and bHLH were able to regulate individual flavonoid-related genes at the transcriptional level and cause the accumulation of one or more particular flavonoid derivatives. In this study, we focused on those BBX members that may regulate anthocyanin biosynthesis, however, several genes showed contradicting results at the phylogenetic analysis, qRT-PCR expression level, and the anthocyanin content result, such as *FtBBX8*, *FtBBX10*, *FtBBX15*, *FtBBX17*, *FtBBX24*, and *FtBBX26*. For example, *FtBBX8* and *AtBBX32* were clustered together in the phylogenetic analysis (Fig. 6), but the expression level of *FtBBX8* continuously increased during light treatment, which was consistent with anthocyanin accumulation (Fig. 8). However, due to the complexity of *BBX* gene function, all the candidate *FtBBX* genes need further analysis to verify their functions in the regulation of anthocyanin biosynthesis.

## Supplemental Information

10.7717/peerj.11939/supp-1 Supplemental Information 1 Primer sequences for qRT-PCR analysis.

10.7717/peerj.11939/supp-2 Supplemental Information 2 The measurements of total anthocyanin content.

10.7717/peerj.11939/supp-3 Supplemental Information 3 Raw data for qRT-PCR.

## Additional Information and Declarations

### Data Availability

The following information was supplied regarding data availability: The raw total anthocyanin data and qRT-PCR are available in the Supplemental Files. The sequences are publicly available at NCBI: SRP157461, SRR6068977, SRR6068978, SRR5433734, SRR5433731, SRR5433730 and SRR5433732.
