# Supplementary material for: Genome-wide analysis of BBX gene family in Tartary buckwheat (Fagopyrum tataricum)
Source: PeerJ. 2021 Aug 11;9:e11939. doi: 10.7717/peerj.11939 (PMC8364324; doi:10.7717/peerj.11939)
Supplement: Supplemental Information 1 [file peerj-09-11939-s001.docx]

| Gene | Forward(5’ - 3’) | Reverse(5’ – 3’) |
| --- | --- | --- |
| FtBBX2 | CTCATCAGACCTCAACCCAC | AATCTTCTACTTGCCAGCCT |
| FtBBX3 | ATGCCTAAGTGCGATATTTG | GGTCCTACCCTGACTCCTGT |
| FtBBX6 | CAACCCTATACCTAATCCCA | AACCCTTAGAAGAGTCAAGAAC |
| FtBBX8 | CTGCTTCGCTCTACTGTCCT | CGTCAATATGATTATCCTCCAT |
| FtBBX9 | ATGCGACGTTCCGATCCACC | ACCGACCACATCTTTACTGTTTGA |
| FtBBX10 | GCATTAGAGTGGCTTTGGAC | GAGGACCTTATTGACAGATTTA |
| FtBBX15 | AAGTGCTCTTCCTCGCTGTG | GGTCGTCAATTCTGATGGGT |
| FtBBX17 | CCTCCTCTTCAGATGCTCCTA | CCCACCCAACTCATTATTCG |
| FtBBX23 | TGATTCCTCCATTGCATTAT | ACATATCTCAGGGACGAAAG |
| FtBBX24 | TGAATCTGCCAATAAGAAAGG | AAATGGAACCGTGTTGTATG |
| FtBBX26 | TTTCCGCGTTGTGATATTTG | ATCATCTCCATTGGGCACTT |

Table S1. The qRT-PCR primers used in the present work
